# Characterizing the Complex Modulus of Asphalt Concrete Using a Scanning Laser Doppler Vibrometer

**DOI:** 10.3390/ma12213542

**Published:** 2019-10-29

**Authors:** Navid Hasheminejad, Cedric Vuye, Alexandros Margaritis, Wim Van den bergh, Joris Dirckx, Steve Vanlanduit

**Affiliations:** 1 Faculty of Applied Engineering, University of Antwerp, 2020 Antwerp, Belgium; cedric.vuye@uantwerpen.be (C.V.); alexandros.margaritis@uantwerpen.be (A.M.); wim.vandenbergh@uantwerpen.be (W.V.d.b.); steve.vanlanduit@uantwerpen.be (S.V.); 2 Laboratory of Biomedical Physics, University of Antwerp, 2020 Antwerp, Belgium; joris.dirckx@uantwerpen.be

**Keywords:** asphalt mixtures, complex modulus, non-destructive testing, laser Doppler vibrometry, modal analysis, master curve

## Abstract

Asphalt mixtures are the most common types of pavement material used in the world. Characterizing the mechanical behavior of these complex materials is essential in durable, cost-effective, and sustainable pavement design. One of the important properties of asphalt mixtures is the complex modulus of elasticity. This parameter can be determined using different standardized methods, which are often expensive, complex to perform, and sensitive to the experimental setup. Therefore, recently, there has been considerable interest in developing new, easier, and more comprehensive techniques to investigate the mechanical properties of asphalt. The main objective of this research is to develop an alternative method based on an optical measurement technique (laser Doppler vibrometry). To do this, a frequency domain system identification technique based on analytical formulas (Timoshenko’s beam theory) is used to determine the complex modulus of asphalt concrete at its natural frequencies and to form their master curve. The master curve plotted by this method is compared with the master curve obtained from the standard four-point bending test, and it is concluded that the proposed method is able to produce a master curve similar to the master curve of the standard method. Therefore, the proposed method has the potential to replace the standard stiffness tests. Furthermore, the standard stiffness methods usually conduct experiments up to the maximum frequency of 30 Hz. However, the proposed method can provide accurate complex modulus at high frequencies. This makes an accurate comparison between the properties of the asphalt mixtures in high frequencies and the development of more accurate theoretical models for simulation of specimens possible.

## 1. Introduction

In traditional structural pavement design, most attention went to the empirical values of material properties in order to design the pavement for certain traffic and climate conditions. However, nowadays, there is a huge interest in understanding the fundamental mechanical properties of asphalt mixtures [1,2] to design optimized roads with longer service life and lower maintenance costs [3]. The first step to accomplish this is to have a good understanding of the mechanical properties of asphalt mixtures.

Assuming asphalt mixtures as being homogeneous, isotropic, and linearly viscoelastic makes it possible to predict their mechanical behavior with two main parameters: complex modulus of elasticity or $E^{*}\left(\omega ,T\right)$ and complex Poisson’s ratio or $\nu^{*}\left(\omega ,T\right)$. $E^{*}$ is the more important parameter of the two and the European standard EN 12697-26:2018 introduces different methods for its characterization [4]. These methods include bending tests and direct or indirect tensile tests performed on asphalt mixtures with different shapes (prismatic, trapezoidal, or cylindrical) under cyclic sinusoidal or haversine loading. By calculating the stress–strain ratio in these experiments, it is possible to compute the complex modulus of elasticity of the materials

(1) $$E^{*}=E^{\prime }+iE^{\prime \prime }=\left|E^{*}\right|e^{i\delta },$$

where $E^{\prime }$ is the storage modulus, $E^{\prime \prime }$ is the loss modulus, and δ is the phase angle.

The traditional standard procedures to obtain the master curve of asphalt mixtures have relatively complicated and expensive experimental setups. An alternative to the standard methods is a modal experiment, which is widely used in many other fields of material characterization and can be applied on a viscoelastic specimen to measure its complex modulus of elasticity. Different methods have been developed based on modal experiments to characterize the properties of asphalt mixtures as well.

For instance, using the fundamental resonance frequencies of asphalt specimens and simplified analytical formulations, it is possible to estimate the complex moduli at different temperatures [5,6]. Although, by having one complex modulus per temperature, it is not possible to form a master curve.

In other research, the first two or three resonance frequencies of cylindrical disk-shaped asphalt samples were found using an accelerometer to measure the vibration caused by the impact of a steel sphere [7] or a hammer [8,9]. Then, by using analytical formulas, the complex moduli of the samples were estimated at those frequencies for multiple temperatures. These methods were able to predict the master curve of asphalt specimens with good accuracy in some frequencies. However, by conducting measurements on a single point, only the first few natural frequencies of the specimens were detected. By having only these natural frequencies of the specimen at each temperature, the experiment had to be repeated at many different temperatures (eleven in one research [8]) to be able to form a full master curve.

Another method to estimate the master curve of asphalt mixtures is the back-calculation technique. In this method, a finite element model (FEM) is used to model the specimen, and the simulations are compared with the experiments. This method has been used to estimate the master curve of an asphalt mixture by minimizing the distances between multiple points on the frequency response functions (FRF) of FEM and experiments [10,11]. This method is extremely time-consuming, and the master curves derived from this method overestimate the complex modulus of the specimens due to the lower strain levels applied to the specimens with the hammer [12].

In this research, a novel, fully automatic experimental setup combined with a new computational approach is proposed to estimate the master curve of asphalt mixtures. In this method, the specimens are excited with a modal shaker to have full control over the excitation force and the measurements are conducted using a scanning laser Doppler vibrometer (SLDV) to have vibration information on the full surface of the specimen (e.g., 95 measurement points on a beam). By doing this, it is possible to compute the mode shapes of the systems and only use the modes that have good quality and match well with the mode shapes acquired from FEM or analytical formulas. Using this method, it was possible to estimate multiple natural frequencies of the specimens (up to 10) at each temperature, which helped to form a master curve with a higher quality with respect to the other methods based on modal experiments. Furthermore, this method needs a less complex experimental setup, is easier to perform, and is cheaper than the standard stiffness experiments.

This paper is outlined further as follows. Section 2 provides the theoretical backgrounds, data processing techniques, procedure of the material production, and experimental setup of the proposed forward-calculation method. The results of these experiments are reported in Section 3 and compared with the results obtained from the standard method. The conclusions of this research finalize this paper in Section 4.

## 2. Materials and Methods

In this section, first, the theoretical background of the methods used in this research are presented. Then the specimen preparation, experimental setup, and the practical implementation of the proposed method are demonstrated.

### 2.1. Theoretical Background

#### 2.1.1. Modal Analysis

Modal analysis is a popular method in many engineering domains, for measuring, improving, and optimizing dynamic characteristics of structures. Modal analysis is the process of determining the dynamic characteristics of a system by its natural frequencies, damping ratios, and mode shapes. These three are called the modal parameters of the system [13].

The idea of modal analysis is to establish a relationship between the excitation force at one location and the vibration response at the same or another location as a function of the frequency. This relationship, which is often a complex mathematical function is the FRF of the system. This excitation is normally produced by either an impact hammer or one or more shakers. Both instruments can produce the required force at different frequencies and measure it using a force transducer. Then, one or more accelerometers are attached to the structure to measure the vibration and form the FRF. Many researchers have already shown that the accelerometer mass loading generates uncertainties in the modal parameters estimation, especially in light or small structures or in highly damped non-linear materials [14]. Therefore, using a non-contact measurement system such as SLDV for vibration measurements at different points of the structure could be beneficial. During the course of this research, an SLDV was used in combination with a shaker to run the modal analysis experiments.

#### 2.1.2. Laser Doppler Vibrometry

The laser Doppler vibrometer (LDV) is a non-contact optical measurement system that operates by measuring the velocity of a point on the surface of an object. LDVs significantly extend measurement capabilities with respect to traditional vibration sensors such as accelerometers. They are able to measure the vibration with a high resolution for an extensive frequency range (reaching more than 1.2 GHz) [15]. Also, since they are not attached to the structure, they can avoid the error caused by the mass loading of the sensors especially in light or small structures or in highly damped non-linear materials such as asphalt mixtures [16].

The improved model of LDV, a scanning LDV or SLDV, has the ability to rapidly and precisely direct the laser to the desired measurement points on the structure using two computer-controlled mirrors. This allows measurements on a predefined grid on a (large) structure with a high spatial resolution. In recent years, the advantages of SLDV have made it a suitable substitution for accelerometers in modal analysis of structures [16,17]. The ability of these instruments to conduct measurements on pavement has been proven in the literature [18,19].

#### 2.1.3. Classical Beam Theories

A beam is a structure where the axial dimension (*L*) is predominant compared to the other dimensions orthogonal to it. The cross-section of the beam is the plane orthogonal to the axis of the structure. There are different models to describe the mechanical behavior of a beam under bending [20]. Two of the popular classic theories are the *Euler–Bernoulli* [21] and *Timoshenko* models [22,23].

The *Euler–Bernoulli* beam equation ignore the effects of shear deformation and rotary inertia and is only accurate for thin beams. In practice, *Euler–Bernoulli* equation is only used to model the first few resonance frequencies associated with the simple mode shapes of a slender beam [24].

Unlike the *Euler–Bernoulli* model, the *Timoshenko’s* beam theory considers the effects of shear deformation and rotary inertia [20], so it is reasonable to assume that it is an improvement of the *Euler–Bernoulli* model [25]. This assumption leads to a more complex procedure to estimate the Young’s modulus of the beam. In summary, by simplifying the FRF equation, part of the denominator of the FRF can be written as $g^{Timo}\left(s\right)$, presented in Equation (2). The full procedure of deriving this formula from the FRF equation can be found in the literature [26].

(2) $$g^{Timo}\left(s\right)=\cosh\left(b_{1}\left(s\right)L\right)\cos\left(b_{2}\left(s\right)L\right)-1+\frac{b_{2}^{2}\left(s\right)-b_{1}^{2}\left(s\right)}{2b_{1}\left(s\right)b_{2}\left(s\right)}\sinh\left(b_{1}\left(s\right)L\right)\sin\left(b_{2}\left(s\right)L\right),$$

where $b_{1}$ and $b_{2}$ are computed using Equation (3):

(3) $$b_{i}^{2}\left(s\right)={\left(-1\right)}^{i+1}c\left(s\right)+\sqrt{c^{2}\left(s\right)+a_{t}\left(s\right)}.$$

The values of c(s) and $a_{t}\left(s\right)$ at pole *s* are given by

(4) $$c\left(s\right)=\frac{\rho }{2E(s)}\left(1+\gamma \left(s\right)\right)s^{2},$$

(5) $$a_{t}\left(s\right)=a\left(s\right)-\frac{\rho^{2}\gamma \left(s\right)}{E^{2}\left(s\right)}s^{4},$$

with a and γ:

(6) $$a\left(s\right)=-\frac{\rho As^{2}}{EI},$$

(7) γ(s)=(12+11ν(s))/5,

where ρ is the density, *A* is the cross-section area ($A=h_{z}h_{y}$ for a rectangular cross-section), and ν is the Poisson’s ratio of the material.

The poles $s_{k}$, which satisfy Equation (8), form an implicit relationship between the resonance frequencies and the material properties of the object:

(8) $$g^{Timo}\left(s\right)=0.$$

This equation is a non-linear algebraic equation and can be solved via the *Newton–Raphson* root finding algorithm:

(9) $$x_{i+1}=x_{i}-f\left(x_{i}\right)/f^{\prime }\left(x_{i}\right).$$

In the *Newton–Raphson* method, first, an initial guess ($x_{0}$,$f(x_{0})$) is assumed for the material. This initial guess, in this case, could be the modulus of elasticity derived from the Euler–Bernoulli method or based on the literature. Next, using these values and the derivative $f^{\prime }\left(x_{0}\right)$, a better approximation for the root is computed. This process is repeated as many times as necessary based on Equation (9) to get the desired accuracy [27].

#### 2.1.4. Master Curve of Complex Modulus

**Figure 1:**
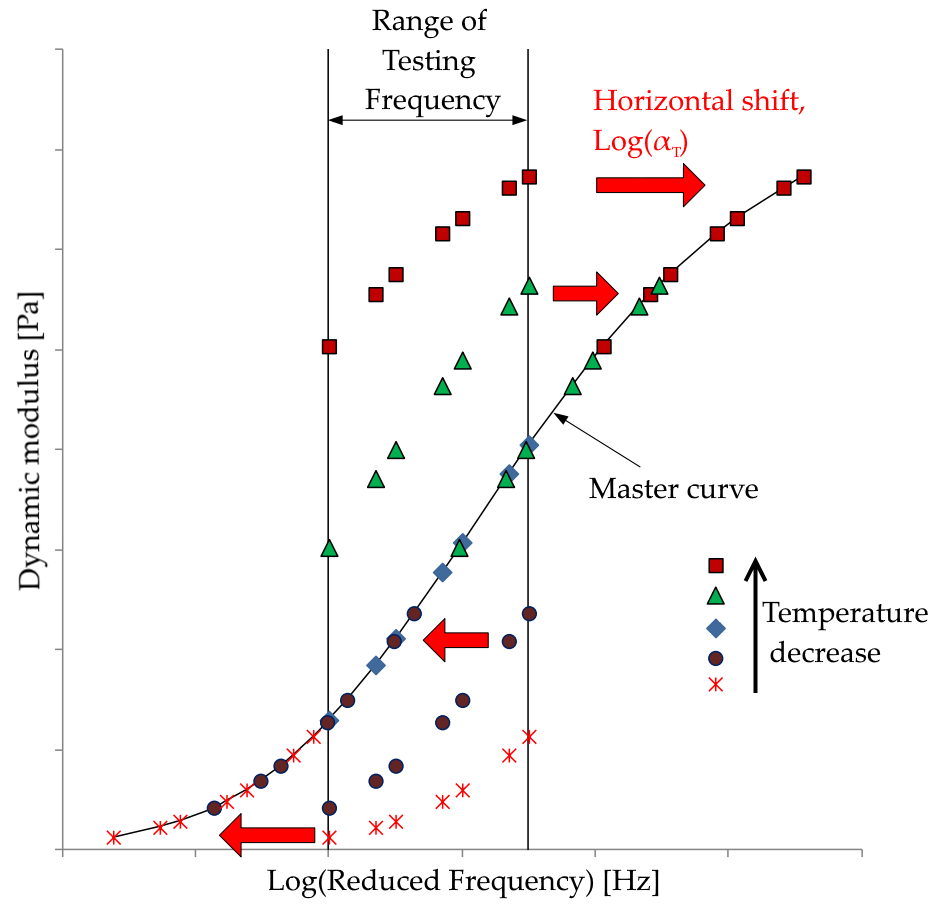
Concept of time–temperature superposition (TTS) principle and master curve formation, each color represents data in different temperatures, and how the data of different temperatures are shifted to form a common curve, called the master curve (the figure is reproduced from [30]).

The mechanical properties of viscoelastic materials such as asphalt mixtures are dependent on the temperature and loading frequency. In order to be able to compare the properties of various mixes, one of these variables has to be normalized. For instance, the data collected at different temperatures can be shifted relative to the loading frequency, so that the various curves at different temperatures can be aligned to form a single master curve. This master curve can later be used to read the stiffness of the material at any specific condition. The technique of the determination of the master curve is based on the time–temperature superposition (TTS) principle. With the TTS principle it is possible to combine the effects of time and temperature for viscoelastic materials. The validity of the TTS principle for different linearly viscoelastic materials, including asphalt mixtures, has been proven by different theoretical and experimental research [28,29]. In this method, a reference temperature ($T_{ref}$) is chosen, and the frequency–modulus of elasticity curves at different temperatures are shifted to form one single master curve (see Figure 1). This frequency shift happens based on a shifting factor $\alpha_{t}$:

(10) $$\log\xi =\log f+\log\alpha_{T}$$

or

(11) $$\xi =f\;\alpha_{T}$$

where ξ is the reduced frequency in Hz, *f* is the loading frequency in Hz, and $\alpha_{t}$ is the shifting factor.

There are different methods to determine the shifting factor $\alpha_{t}$ [31,32]. In this research, the *Williams–Landel–Ferry* (WLF) [33] method is used for its simplicity and ability to provide satisfactory results. The WLF equation is as follows:

(12) $$\log\alpha_{T}=-\frac{C_{1}\left(T-T_{ref}\right)}{C_{2}+T-T_{ref}},$$

where $C_{1}$ and $C_{2}$ are the empirical values determined from experiments.

A popular mathematical model to construct the master curve of asphalt mixtures is the *sigmoidal fitting function* (see Equation (13)). The advantage of sigmoidal functions over the polynomial functions is their ability to model the curve better at low and high temperatures [34].

(13) $$\log(|E^{*}\left|\right)=S_{min}+\frac{\alpha }{1+e^{\beta -\gamma \log(\xi )}},$$

where $S_{min}$ is the minimum modulus value, α is the span of the modulus value, and β and γ are shape parameters.

To simplify the coding and calculations to form the master curve, in this research, Equation (13) is written as [35]:

(14) $$\log(|E^{*}\left|\right)=\log\left(S_{min}\right)+\left[\log\left(S_{max}\right)-\log\left(S_{min}\right)\right]S,$$

with

(15) $$S=1-\exp[-{\left(\frac{10+\log\xi }{\beta }\right)}^{\gamma }],$$

where $S_{min}$ and $S_{max}$ are the minimum and maximum stiffness in MPa respectively.

In this research, this procedure is used twice. First, the results of the standard four-point bending test on prismatic specimens (4PB-PR) are analyzed according to these steps, and the six unknown parameters of the sigmoidal function and WLF equations are estimated. Second, the estimated $|E^{*}|$ of the proposed forward-calculation method are used to optimize the six unknown parameters. The complete procedure of data processing for both methods are presented in Section 2.2.3, and the optimized parameters are presented in Section 3.2.

### 2.2. Practical Implementation

#### 2.2.1. Material Production

**Figure 2:**
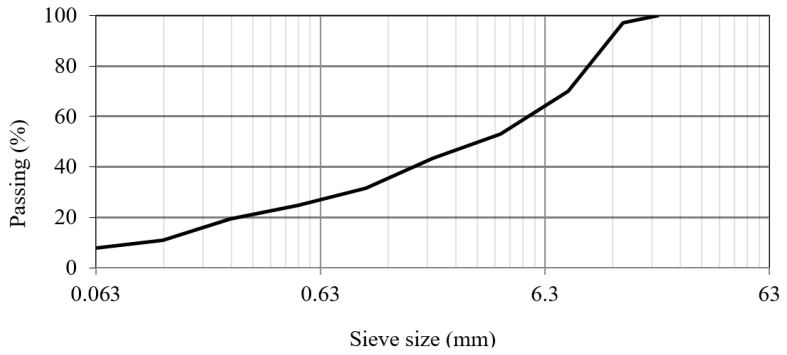
Grading curve of the designed mixture.

In this study, all the experiments were conducted on a typical base layer mixture developed in the EMIB research group as a reference for common tests. This mixture has a maximum aggregate size of 14 mm (AC14) and contains 4.3% of bitumen by mass of aggregates. A common 35/50 penetration grade bitumen is used. Table 1 shows the basic properties of the used bitumen. These properties are based on the datasheets of the supplier and confirmed by lab tests according to the standards NBN EN 1426:2015 [36], NBN EN 1427:2015 [37], and NBN EN 12593:2015 [38]. The composition of the mixtures is presented in Table 2 and Figure 2 shows the final grading curve of the mixture.

In total, six prismatic beams were prepared. Three (B1–B3) for the modal analysis experiments and three (R1–R3) for the reference four-point bending test. Since the dimensions of the specimens do not influence their mechanical properties, the dimensions of the samples of each experiment are chosen based on the restrictions of the experimental setups. The dimensions of B1–B3 specimens were chosen (see Table 3) considering these factors:

1. The specimens are beam shaped to follow the classical beam theories (stated in Section 2.1.3).
2. According to NBN EN 12697-26:2018 [4], for the asphalt specimens to represent their true material properties, it is recommended that their width $h_{y}$ and height $h_{z}$ be at least three times the maximum grain size of the mixture. Therefore, the width $h_{y}$ and height $h_{z}$ of the beam for this mixture are higher than 42 mm.
3. The samples should fit in the available climate chamber, so in this study, their length *L* should be less than 45 cm, which is the width of the frame built to be placed inside the climate chamber (see Figure 4b).

**Figure 3:**
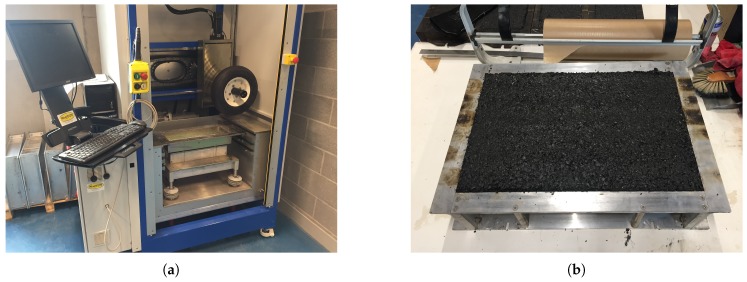
(**a**) The large scale roller compactor, and (**b**) the manufactured asphalt concrete in the mold.

Furthermore, the dimensions of the reference specimens R1 to R3 are in accordance with NBN EN 12697-26:2018 [4] and presented in Table 3. The three B1 to B3 beams were produced and cut from two plates (P1 and P2) at the asphalt lab of the University of Antwerp, and the three R1 to R3 beams were cut from one plate (P3). The production of P3 was done at Agentschap Wegen en Verkeer (Translation: Flemish Agency for Roads and Traffic) (AWV), and it was cut at the asphalt lab of the University of Delft, as part of the experimental equipment was temporarily out-of-order. The plates were produced with the same procedure aiming at similar volumetric properties, including air voids, maximum density and bulk density. The mixture was manufactured according to NBN EN 12697-35:2016 [39] and compacted with a large scale roller compactor using the 2-wheel heavy compaction method, according to NBN EN 12697-33+A1:2007 [40]. The mixing temperature was 175 °C and the compaction temperature 165 °C (see Figure 3).

#### 2.2.2. Experimental Setup

**Figure 4:**
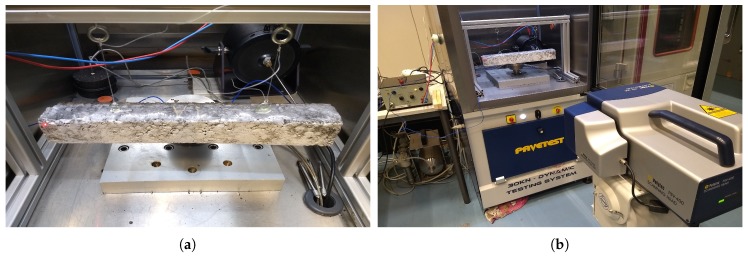
Experimental setup: (**a**) The shaker exciting the beam-shaped asphalt mixture from behind; (**b**) the scanning laser Doppler vibrometer (SLDV) conducting vibration measurements on the surface of the object inside the climate chamber. This modal experiment was conducted in five temperatures and the results are the inputs of the proposed methods for material properties estimation.

After the preparation of beams B1 to B3, their front sides were painted by the white spray paint (Ardrox^®^ 9D1B aerosol by Chemetall, Frankfurt am Main, Germany) to improve the LDV measurement quality as suggested in the literature [19]. Next, the specimens were suspended from a small frame built for the climate chamber of the Universal Testing Machine (UTM) using two screw eyes and fishing lines to simulate free-free conditions. The experiments were conducted at five temperatures (5, 10, 15, 20, and 30 °C). These temperatures were chosen in a way that they cover a wide range of the master curve while taking into account the operational temperature of the shaker, which cannot go lower than 0 °C or exceed 40 °C. The shaker was a Vibration Exciter type 4809 by Brüel & Kjær, Nærum, Denmark, exciting the specimens with a periodic chirp signal between the frequency range of 800 to 19,200 Hz (see Figure 4a). Signals were generated using the Polytec onboard signal generator and amplified by a Brüel & Kjær power amplifier type 2706. A Brüel & Kjær force transducer type 8230-001 was placed between the tip of the stinger of the shaker and the specimen to measure the force, and a He-Ne SLDV (PSV-400 by Polytec, Waldbronn, Germany) was used to measure the vibration velocity of 95 scanning points on the surface of the specimens (see Figure 4b).

#### 2.2.3. Data Processing

**Figure 5:**
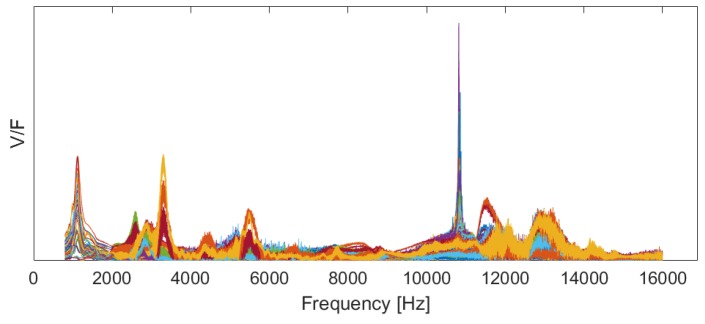
Frequency response functions (FRF) of the 95 scanning points of the specimen B3 at 5 °C as an example. (V/F is the velocity of the scanning points over the excitation force measured by the force transducer).

At this stage, using the velocity of the scanning points, measured by the SLDV, and the measured excitation force, the FRF of the samples B1–B3 at each scanning point is calculated (see Figure 5). To increase the quality of the measurements, each measurement is conducted five times on each of the scanning points. This makes the formation of the coherence function possible to investigate the repeatability of the measurements.

The FRFs (presented in Figure 5) are the input to the the PolyMAX or polyreference least-squares complex frequency-domain method [41] that uses a least-squares approach to fit a rational fraction polynomial model to the FRFs. The PolyMAX estimator uses the FRFs of the structure as primary inputs and is able to estimate the modal parameters, including the natural frequencies, damping ratios, and the mode shapes of the system [42]. The poles of the system $s_{k}$ can be found from this algorithm and are in relation with eigenfrequencies $\omega_{k}$ and damping ratios $\xi_{k}$:

(16) $$s_{k}=-\xi_{k}\pm i\sqrt{1-\xi_{k}^{2}\omega_{k}}.$$

**Figure 6:**
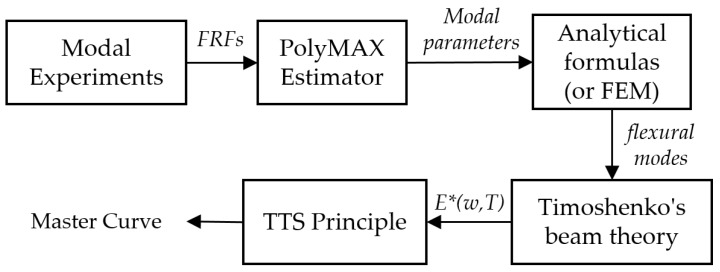
Overview of the proposed forward-calculation method.

Next, the mode shapes are plotted for each pole of the system. By comparing these mode shapes with the mode shapes derived from a FEM (a simple beam modeled in COMSOL Multiphysics) using the modal assurance criterion (MAC), the flexural mode shapes of the specimens are separated automatically. However, it is also possible to use analytical formulas instead of a FEM to separate the flexural mode shapes of the samples. Then, as explained in Section 2.1.3, the Timoshenko’s beam theory is used to compute the complex moduli of the specimen at different temperatures and natural frequencies. Finally, using the TTS principle presented in Section 2.1.4, the magnitude of the complex moduli are shifted to form the master curve of the specimen. Figure 6 illustrates an overview of the procedure to obtain the master curve from modal analysis experiments.

## 3. Results and Discussion

### 3.1. Repeatability of the Measurements

**Figure 7:**
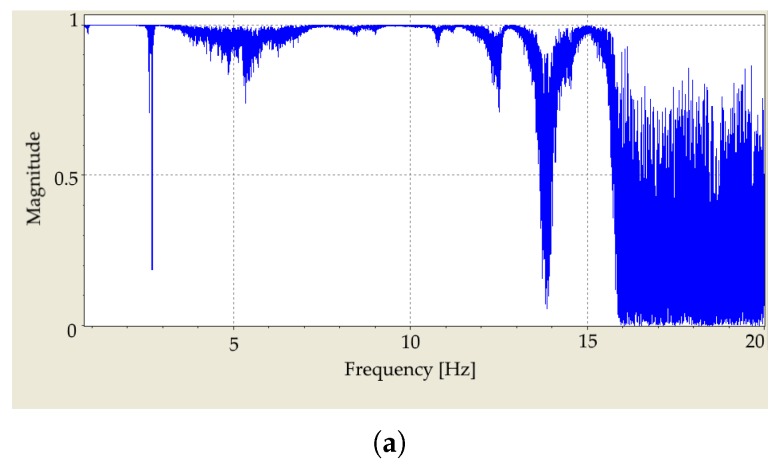

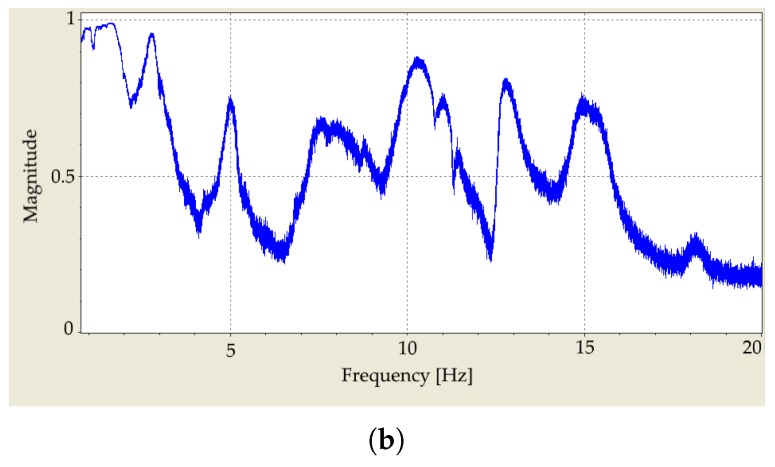
(**a**) Coherence function of the FRF based on five measurements on a single point on the surface of the asphalt specimen. (**b**) Averaged coherence function of FRF of 95 grid points (five measurements per point). Values close to 1 in the top figure show the good repeatability of the measurement up to 15 kHz. For higher frequencies, the excitation force is inadequate to excite the specimen sufficiently. The lower value of the coherence function in the second figure illustrates that an asphalt mixture is a challenging material to measure and model.

**Figure 8:**
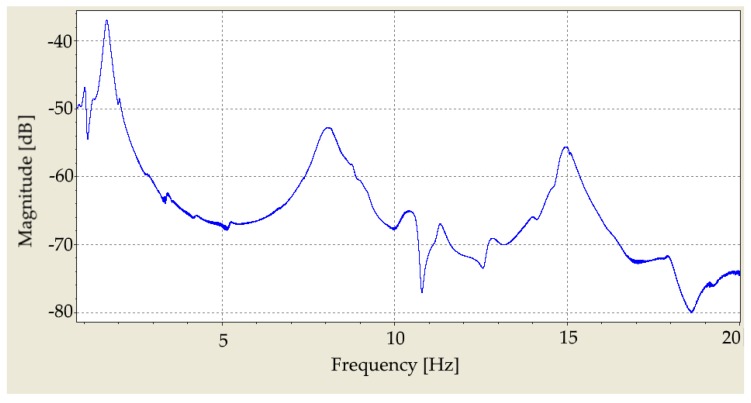
Excitation force applied by the modal shaker and measured with a force transducer placed in between the tip of the stinger and the specimen (0 dB = 1 V). It can be seen that the force level is relatively low near the natural frequencies and in high frequencies.

Figure 7a illustrates the coherence function computed for a single point on the surface of the specimen. It can be seen that the coherence is close to 1 until 15 kHz and then it decreases. The reason is the low excitation force at high frequencies, as presented in Figure 8. Although measurements with SLDV on multiple points of the asphalt surface is challenging. This leads to lower averaged coherence function for the 95 measurement points on the surface of the specimen as presented in Figure 7b.

### 3.2. Complex Modulus of Elasticity

Table 4 presents the natural frequencies of the specimens ($f_{k}=\omega_{k}/2\pi$) and the percentage of damping ratios ($D_{k}=\xi_{k}\times 100$) for the modes with a MAC value above 75%. The reason to choose a relatively low MAC limit was the fact that the grids of the FEM and the scanning grid of the experiments were matched by manually measuring the location of three points on the surface of the specimen and then defining them in the FEM. The accuracy of this method is dependent on the measurements done by hand but provided enough information to match the similar mode shapes of the experiments with the FEM. Table 4 only contains the flexural mode shapes of the specimens since only those mode shapes are used in the Timoshenko’s beam theory to estimate the complex modulus.

In this table it is shown that increasing the temperature decreases $|E^{*}|$ for all frequencies, as expected. Furthermore, at a specific temperature, $|E^{*}|$ is higher at higher natural frequencies. These two observations are in accordance with the behavior of the viscoelastic materials such as asphalt mixtures in the literature [4,43].

After the calculation of the complex stiffness modulus for each flexural mode shape (Table 4), the six variables of the Sigmoidal function (β, γ, $S_{min}$, and $S_{max}$) and the shifting parameters ($C_{1}$ and $C_{2}$) are optimized for the B1–B3 specimens with the method explained in Section 2.1.4. These results are shown in Table 5.

To have a reference master curve, 4PB-PR experiments were conducted, and the amplitude of the complex stiffness modulus measured by the UTM at four temperatures (−15, 0, 15, 30 °C) and eight frequencies (0.1, 0.2, 0.5, 1, 2, 5, 10, 20 Hz) were exported to MATLAB. Then, using the procedure explained in Section 2.1.4, the parameters of the master curves of the R1–R3 specimens and the averaged data were optimized and presented in Table 5.

### 3.3. Master Curves Estimated by the Proposed Forward-Calculation Method

**Figure 9:**
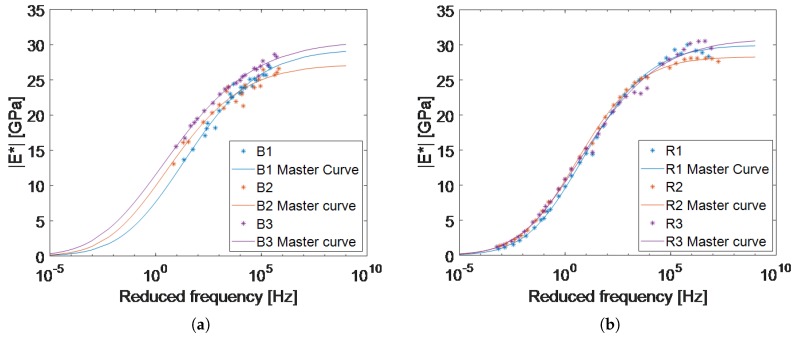
(**a**) Master curves of the B1–B3 specimens at 15 °C estimated using modal analysis and Timoshenko’s beam theory. Specimen B3 was cut from a different asphalt plate than specimens B1 and B2. That could be the reason it has a slightly higher $|E^{*}|$ in all frequencies. (**b**) Master curves of the reference specimens R1–R3 based on the 4PB-PR method at 15 °C. It can be seen that the three curves are not a perfect match even using the standard method.

Using the parameters presented in Table 5, the master curves of the three B1–B3 samples, and the three reference samples (R1–R3) were computed with the proposed forward-calculation and the standard methods respectively (Figure 9). It is evident that the master curves of the forward-calculation method have higher variations at different frequencies in comparison with the master curves calculated from the 4PB-PR tests. The reason could be that the specimens of the 4PB-PR test (R1–R3) were all cut from a single plate, but the B1–B3 samples were cut from two different plates, which were compacted at different facilities. Furthermore, the R1–R3 samples were cut on all their six faces, but to prove that the SLDV used in this chapter is able to obtain results from the rough surface of the asphalt, only the top and bottom faces of the B1–B3 samples were cut.

Figure 9a illustrates that the measurement results, on which the master curves of the forward-calculation method are based, are located in the frequency range of 7 Hz to 0.7 MHz. This is due to certain limitations considering the operational temperature of the shaker used in the experiments (0 °C to 40 °C) and the width of the climate chamber (45 cm). This limited frequency band can get wider by conducting experiments at a more comprehensive temperature range or using longer beams. This will decrease the first natural frequency of the specimens, which will lead to the measurement of more natural frequencies and hence more complex modulus points will be available to calculate the master curve.

Besides all the limitations mentioned above, Table 5 shows that there is a good correlation between the average of the parameters computed based on the two methods. For instance, $S_{max}$, which shows the limit of the maximum $|E^{*}|$ at high frequencies, shows only 2.3% difference between the two methods. This difference is lower than the standard deviation for both methods. Comparing the other parameters one by one is not easy, and it is more convenient to compare the master curves produced by all the parameters together. For instance the value of $S_{min}$ calculated for B1–B3 is 66.9% different than that of R1–R3. However, this does not matter since this value plays only a small role in the final master curve, in the desired frequency range. In general, other than parameters β and $S_{max}$, the standard deviation of the parameters calculated with the proposed method are lower than that of the standard method.

**Figure 10:**
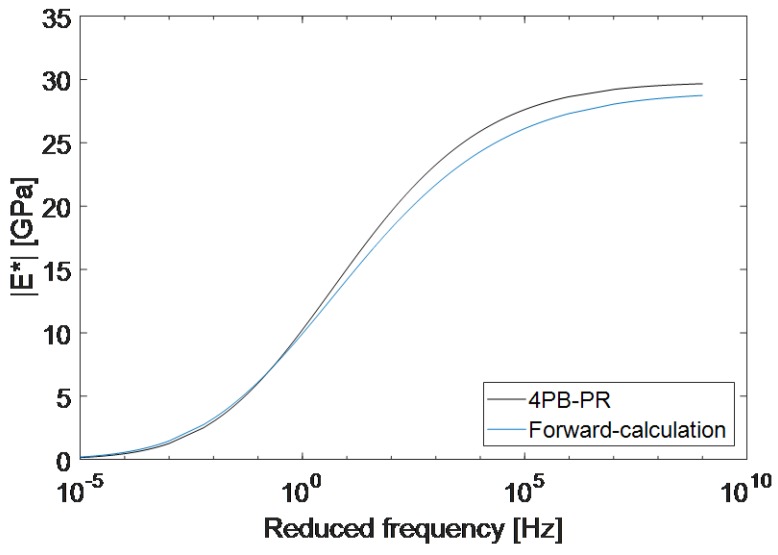
Comparison between the master curve achieved from the reference four-point bending test and the master curve acquired from the Timoshenko’s beam theory at the reference temperature of 15 °C.

Finally, using the averaged parameters from Table 5, the master curves derived with both methods are plotted in Figure 10. It can be seen that the two average master curves show a very good correlation with each other. The difference between the two master curves is 0.09 GPa at 0.1 Hz and increases to a maximum of 1.62 GPa at 100 kHz and decreases to 0.9 GPa at 1 GHz. At higher frequencies, a slightly lower absolute value for the complex moduli was found compared to the standard method. This is most likely due to the differences in the samples produced for each method and acceptable when conducting experiments on complex materials such as asphalt mixtures.

**Figure 11:**
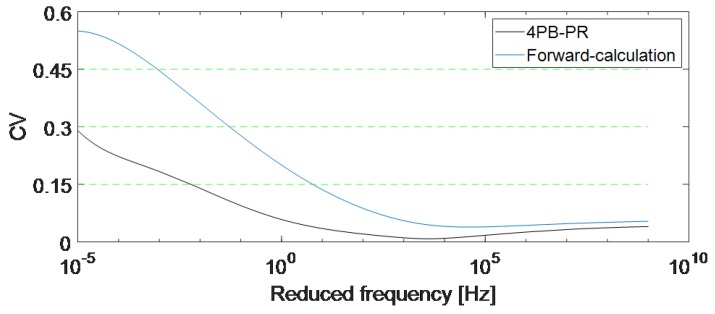
Comparison of the coefficient of variation (CV) of the master curves achieved from two methods.

To check the repeatability of the experiments, the coefficient of variation (CV) is computed. CV is the standard deviation over mean and according to the literature, it is normally between 0.15 and 0.30 when doing material tests on asphalt mixtures. CV = 0.15, 0.3, and 0.45 mean low variation, high variation, and extremely high variation between the measurements, respectively [44]. CV is especially important between $10^{-3}$ Hz to $10^{7}$ Hz, which, considering the temperature shift, is the range that must be measured according to the European standards [4]. Figure 11 shows that in this range, the CV of the 4PB-PR experiments is between 0.18 and 0.032, meaning an acceptable deviation between the master curves of R1–R3 samples. The CV of the forward-calculation method is higher than the CV of the 4PB-PR experiments in this range. The reason for this high variation is the fact that the master curves are not based on measured $|E^{*}|$ at low frequencies and are extrapolated (see Figure 9a). Furthermore, as explained before, the specimens of the 4PB-PR method were cut from one plate, but the specimens of the forward-calculation method were cut from two plates which can increase the CV as well.

### 3.4. Advantages and Limitations of the Proposed Method

The first advantage of the proposed forward-calculation method over the traditional method is the less strict limitations on the dimensions of the testing samples. For instance, according to the 4PB-PR test conducted with the available UTM for this research, the length of the beam must be between 350 mm and 462 mm. However, this limitation does not exist in the forward-calculation method.

Furthermore, the cost of the equipment used in the forward-calculation method is competitive with a basic stiffness testing system to conduct the standard experiments. A simple testing machine able to run stiffness tests according to the indirect tensile testing standard costs about 215 k€. On the other hand, the testing setup proposed in this article (including an SLDV, a modal shaker, an amplifier, and a climate chamber) approximately costs 170 k€. It should be mentioned that, as explained in Section 2.1.2, SLDVs are applicable in many different fields and can be used for other experiments as well. Furthermore, sample preparation and testing time of the methods are similar. The experimental settings and the equipment used in the forward-calculation experiments are less complex and conducting the tests is less complicated. However, the computational time and the post-processing of the forward-calculation method is currently a few minutes longer than the computational time of the standard method.

The main practical limitations of the method presented in this research were:

1. The operational temperature of the shaker used in this research was between 0 to 40 °C. Therefore, the testing temperatures were selected based on this limitation. By using a shaker with a wider operational temperature range or designing a climate chamber in a way that the shaker can be placed outside of the chamber, it is possible to conduct measurements in a wider temperature range. This leads to more data for master curve creation and therefore, more accurate master curves.
2. The first natural frequency of the beam in this study was relatively high. Therefore the first point of the master curve in 15 °C was located at 7 Hz, and the master curve was extrapolated for lower frequencies. This could cause a less accurate result in low frequencies. This problem can be solved by increasing the length of the beam or decreasing the cross-section dimensions. As explained before, the cross-section of the sample cannot be smaller than three times the biggest aggregate in the mixture. Therefore for this base layer mixture, the lowest cross-section could be 4.2 mm. Furthermore, the length of the beam was selected based on the dimensions of the available climate chambers. By using a larger climate chamber, it is possible to produce longer specimens with a lower first natural frequency. In that case, more data will be available at lower frequencies which is beneficial for the formation of a more accurate master curve. For instance, according to the analysis done by FEM, the first natural frequency of the asphalt mixture used in this research with dimensions of 0.6 m ×0.042 m ×0.042 m is 193 Hz, which considering the shifting parameters, means a data point at 0.5 Hz for the master curve plotted in a reference temperature of 15 °C. Producing specimens with this dimension is convenient in the asphalt lab since the asphalt plates are normally produced with 0.6 m ×0.4 m $\times \;h_{z}$ m dimension and the beams with the proper cross-section can be cut from them.
3. According to the datasheets of the shaker used in this research, the frequency range of the shaker is 10 Hz to 20 kHz. However, in these experiments, the shaker was not able to sufficiently excite the specimen in high frequencies, which led to noisy data at some frequencies. Therefore, the mode shapes plotted at high frequencies did not match very well with the FEM model causing low MAC values, so they were removed from the calculations. Having a more powerful shaker can lead to acquiring more mode shapes of the system and therefore, estimation of more complex moduli and more accurate master curves. However, it is also essential to be careful not to increase the load too much, to keep the strain under the level in which the material can be considered as linear viscoelastic.

## 4. Conclusions

In this research, a forward-calculation procedure is proposed to calculate the complex modulus of asphalt mixtures and form their master curves. First, a modal analysis experiment was designed using a shaker and scanning laser Doppler vibrometer to conduct modal experiments on asphalt specimens suspended in free-free condition. Then the results of these experiments were analyzed by a PolyMAX estimator to estimate the modal parameters of the specimens. Afterwards, the Timoshenko’s beam theory was used to find the complex modulus of the specimens at different temperatures and natural frequencies. These complex moduli were used to form the master curve of an asphalt mixture using the Sigmoidal function. Finally, it was shown that the master curves calculated by the proposed forward-calculation method correlate with the master curves plotted using the traditional 4PB-PR tests. This method seems promising in characterizing the linear viscoelastic behavior of asphalt mixtures by producing an accurate master curve of $|E^{*}|$ and has the potential to be used instead of the traditional stiffness tests. Since the standard stiffness methods used today are limited to testing at a maximum frequency of 30 Hz, the proposed forward-calculation method can provide more accurate knowledge about the complex modulus of the asphalt mixture at high frequencies and low temperatures. This information can be used for accurate comparison between the properties of the asphalt mixtures in high frequencies, and development of more accurate theoretical models for simulation of specimens. Moreover, this method can be used to estimate the complex modulus master curve of other viscoelastic materials such as asphalt mortar, and further research to explore this idea is of interest.

## Figures and Tables

**Table 1:** Basic properties of the 35/50 penetration grade bitumen.

Property (Unit)	Penetration (1/10 mm)	Softening Point (°C)	Fraass Breaking Point (°C)
Value	37	54.3	−10

**Table 2:** Mixture composition (by aggregate mass).

Limestone 6.3/14	39.8%
Limestone 2/6.3	14.0%
Limestone 0/2	30.0%
River sand 0/1	7.5%
Filler (filler 15)	8.7%
Total	100%
Bitumen 35/50	4.3%

**Table 3:** Dimensions of the beams used for modal analysis (B1–B3), and the beams for the reference standard experiments (R1–R3).

Specimen	Height (H, cm)	Width (W, cm)	Length (L, cm)
B1	5.23	5.40	40.0
B2	5.27	5.37	39.9
B3	5.26	5.51	40.0
R1	5.02	5.07	44.8
R2	4.95	5.07	44.8
R3	4.34	5.00	44.7

**Table 4:** Natural frequencies and percentage of the damping ratios of B1, B2, and B3 samples determined from the modal analysis experiments using the PolyMAX estimator and the corresponding complex stiffness modulus at each mode shape computed by the Timoshenko’s beam theory.

T (°C)	Mode Shape #	B1			B2			B3		
		freq. (Hz)	D (%)	|E*| (GPa)	freq. (Hz)	D (%)	|E*| (GPa)	freq. (Hz)	D (%)	|E*| (GPa)
5	1	1080.2	4.7	23.91	1077.4	3.8	23.90	1160.6	3.6	26.64
	3	2809.4	3.2	25.17	2875.2	4.0	26.45	2990.7	3.3	27.68
	4							5224.9	4.5	27.08
	6	7460.6	4.2	25.72						
	8	10,013.5	2.3	25.68	10,023.8	2.5	25.71	10,662.5	1.8	28.61
	10	12,974.6	2.5	27.07	12,741.2	3.0	26.06	13,360.1	2.6	28.28
	12	15,610.2	3.4	26.72	15,594.0	1.7	26.60			
10	1	1041.6	4.7	22.50	1031.9	5.1	21.93	1114.0	4.7	24.55
	3	2775.2	5.9	23.94	2754.0	4.3	24.26	2880.8	4.2	25.68
	6	7369.1	6.0	25.09						
	8	9706.1	3.4	24.13				10,256.3	3.6	26.48
	10				12,503.6	2.6	25.09	12,699.7	1.2	25.55
	12	15,080.7	4.1	24.94	14,848.8	3.2	24.12	15,775.2	3.4	26.93
15	1	1003.2	5.5	20.62	1020.9	5.2	21.46	1077.8	6.2	22.97
	3	2613.9	5.8	21.79	2740.6	4.6	24.03	2782.5	5.0	23.96
	4	4899.1	5.7	24.41	4225.6	1.6				
	8	9500.6	3.6	23.11				9953.8	3.6	24.94
	10	12,026.9	3.8	23.26	11,964.0	6.7	22.97	12,674.5	3.1	25.45
	12	14,763.7	4.9	23.90						
20	1	939.7	10.1	18.09	960.8	10.9	19.01	1020.9	8.6	20.61
	3	2388.3	9.1	18.19	2520.9	7.5	20.33	2649.2	6.4	21.72
	8				9055.9	5.7	20.98	9704.6	6.3	23.70
	10	11,963.4	6.1	23.01	12,072.6	5.5	23.39			
	12	14,352.7	5.7	22.59						
30	1	816.6	15.2	13.66	796.8	13.6	13.07	886.2	14.1	15.53
	3	2177.9	10.0	15.12	2244.2	11.3	16.11	2325.8	14.2	16.74
	6							6469.7	9.7	18.94
	8	8169.3	5.3	17.09				8798.3	8.4	19.48
		10	10,829.9	7.9	18.86					

The gray cells contain the mode shapes with low MAC values that are automatically eliminated from the analysis.

**Table 5:** The optimized values of the parameters of the shifting factor (Equation (12)) and the Sigmoidal function (Equations (14) and (15)) for both four-point bending test on prismatic specimens (4PB-PR) tests (R1–R3 specimens) and the forward-calculation method (B1–B3 samples). Using these values the master curves of the specimens can be plotted. The standard deviation of all the values other than β and $S_{max}$ is higher for reference specimens than the B1–B3 specimens.

Sample	β	γ	C1	C2	Smin (MPa)	Smax (MPa)
**R1**	7.17	2.15	29.47	190.88	4.49	29,960.97
**R2**	7.34	2.29	20.76	134.53	16.95	28,291.33
**R3**	6.34	1.77	31.88	199.93	1.21	30,868.85
**Average**	6.95	2.07	27.37	175.11	7.55	29,707.05
**std**	0.44	0.22	4.78	28.93	6.78	1067.48
**Ave. R1–3 measurements**	6.81	1.98	26.97	172.35	3.39	29,793.11
**B1**	7.28	2.00	19.12	167.06	4.35	29,329.50
**B2**	6.35	1.82	20.91	138.11	1.53	27,186.87
**B3**	5.87	1.57	19.25	130.58	1.63	30,554.48
**Average**	6.50	1.80	19.76	145.25	2.50	29,023.62
**std**	0.59	0.17	0.82	15.73	1.30	1391.73

Ave. R1–3 measurements means that instead of averaging the optimized parameters directly, first the average of the complex moduli of the three specimens at each frequency and temperature is computed and then the parameters are optimized.
